# Employment of Flake Ice Systems Including Natural Preservative Compounds for the Quality Enhancement of Chilled Seafood—A Review

**DOI:** 10.3390/antiox10091499

**Published:** 2021-09-21

**Authors:** Santiago P. Aubourg

**Affiliations:** Department of Food Science and Technology, Marine Research Institute (CSIC), Calle Eduardo Cabello, 6, 36208 Vigo, Spain; saubourg@iim.csic.es; Tel.: +34-9862-31930

**Keywords:** seafood, flake ice, plant extracts, organic acids, algae extracts, by-product extracts, antioxidant, antimicrobial, quality, sensory acceptance

## Abstract

Marine species deteriorate rapidly post-mortem as a consequence of a variety of biochemical and microbial breakdown mechanisms. Due to the increasing demand for high-quality fresh seafood, different strategies are now available to retard spoilage for as long as possible. The present study provides an overview of a recently proposed strategy based on the addition of natural compounds to marine species. In this strategy, different kinds of natural preservative compounds are included in the flake-ice medium that is commonly used for chilled storage. Natural sources tested for this purpose include low-molecular-weight organic acids and different kinds of extracts of plants, macroalgae, and by-products resulting from marine species commercialization. The preservative action of such treatments is analyzed according to the effect on different deteriorative mechanisms (i.e., lipid hydrolysis, oxidation, and microbial activity development), as well as on the resulting sensory acceptability and shelf-life time. The basic objective of this review is to provide an overview concerning the positive effect that the presence in an icing system of natural preserving compounds may have on the quality of chilled marine species. Furthermore, various potential avenues are proposed to develop the practical and commercial employment of this technological strategy.

## 1. Introduction

Marine species are considered to be among the most perishable foods; even when kept under appropriate conditions, the quality quickly deteriorates [1,2]. Loss of quality in fish is brought about initially by autolytic deterioration, due to the action of enzymes that are present in the gut and in the flesh of the fish. This is followed by the growth of microorganisms on the surface of the fish, which manifests itself as a slime developing on the surface. The bacteria then invade the flesh of the fish, causing breakdown of the tissues and a general deterioration of the product. In the case of fatty species, an additional and significant deteriorative mechanism is lipid oxidation; on the basis of the high content of unsaturated fatty acids, a wide range of off-odor and off-flavor molecules can be produced in parallel to autolytic and microbial degradation and lead to substantial quality losses. Generally, the rates at which all deteriorative mechanisms take place are dependent upon the temperature at which the fish is stored [3].

Among marine products, fresh ones have captured the market and represent very high proportions of fish production and human consumption. In order to keep the original properties of marine species, chilling represents the most employed strategy to provide the consumer with high quality seafood [4,5]. The extension of the shelf-life of chilled fish products has been a constant need because such products are often transported and sold at distant markets. Traditionally, chilled fish products have been preserved by packaging with flake ice, to partially inhibit their quality loss. This is still the most widely used tool today. However, problems related to fishing management and shelf-life times when long-storage periods are required have led to the search for alternative technologies [6,7].

In connection with the above-mentioned trend of fresh seafood consumption, public health concerns in the marine food trade have become an issue requiring careful attention to ensure, not only safety, but also sensory acceptability and nutritional value. Consequently, storage under flake ice conditions has been combined with other preservative strategies, such as physical (high-pressure processing, different kinds of irradiation, active and intelligent packaging, etc.) [8,9] and chemical (addition of synthetic and natural preservative compounds) treatments [10,11,12]. In such cases, a combination of strategies has been applied, so that each one provides different and complementary advantages (or barriers to spoilage) for ensuring quality retention.

The present study provides an overview on a recent strategy based on the employment of natural compounds for marine species chilling. In this strategy, different kinds of natural preservative compounds are included in the flake ice medium that is used for the chilling storage. Natural sources tested for this purpose include low-molecular-weight organic acids and different kinds of extracts of plants, macroalgae, and by-products resulting from marine species commercialization. The preservative action of such chilling treatment is evaluated according to the effect on different deteriorative mechanisms (i.e., lipid hydrolysis and oxidation and microbial activity development) as well as on the resulting sensory acceptability and shelf-life time.

## 2. Plant-Extract Presence in the Icing Medium: Effect on Chilled Seafood Quality

### 2.1. General Aspects of Plant-Extract Compounds

Since ancient times, spices and herbs have been added to food as seasoning additives due to their aromatic properties. Nowadays, plant extracts are well known as bio-preservatives, as they have been shown to inhibit the microbial growth of Gram-positive and Gram-negative bacteria, yeasts, and molds, and also exhibit useful antioxidant activity [13,14,15]. Among these, herbs of the *Lamiaceae* family, mainly oregano (*Origanum vulgare*), rosemary (*Rosmarinus officinalis*), and sage (*Salvia officinalis*), have been extensively reported as having significant preservative capacities. Although most of such plant products are classified as generally recognized as safe (GRAS), their use in food as preservatives is limited because of flavor considerations, since effective preservative doses may exceed acceptable sensory levels [16].

Among plant-derived compounds, phenolic volatiles, also known as essential oils (EO), are the main active ingredients in most herbs, e.g., menthol (in mint *Mentha canadensis*), carvacrol (in oregano and rosemary), thymol (in thyme *Thymus vulgaris*), and eugenol (in clove *Syzygium aromaticum*) [17,18]. Thus, the oregano EO contains up to 50% thymol; thyme EO has 43% thymol and 36% p-cymene, and savory EO, 30–45% carvacrol and 30% p-cymene [19,20]. Notably, the main components of EO are terpenoids, specifically monoterpenes (C10) and sesquiterpenes (C15), as well as a variety of low-molecular-weight compounds.

The antimicrobial activity of plant-derived compounds against many different microorganisms, tested individually and in vitro, is well documented in the literature [12,21]. The active compounds responsible for the antimicrobial activity of spices are primarily phenolic components of the EO fraction. Thus, the antimicrobial activity of cinnamon (*Cinnamomum* spp.), allspice (*Pimenta dioica*), and clove (*S. aromaticum*) is attributed to eugenol (2-methoxy-4-allyl phenol) and cinnamic aldehyde, which are major constituents of the volatile oils of these spices. The total antioxidant capacity of fruit and vegetable extracts reflects concentrations of ascorbic acid (vitamin C), alpha-tocopherol (vitamin E), beta-carotene (vitamin A precursor), various flavonoids, and other phenolic compounds [22]. Such compounds have shown the ability to terminate free-radical reactions and scavenge for reactive oxygen species. Among the main phenolic compounds identified in plant extracts, phenolic acids (e.g., p-coumaric acid, caffeic acid, rosmarinic acid, and gallic acid), phenolic diterpenes (e.g., carnosic acid and epirosmanol), and flavonoids (e.g., aromatic compounds) can be mentioned [11,18].

### 2.2. Quality Enhancement of Chilled Seafood by Including Plant Extracts in Ice

Oral et al. [23] analyzed the effect on gutted and ungutted Transcaucasian barb (*Capoeta capoeta capoeta*) of ice produced from an aqueous solution of wild-thyme (*Thymus serpyllum*) hydrosol for 20-day chilled storage. The results obtained did not reveal marked differences in the microbial counts, sensory characteristics, pH, and total volatile basic nitrogen values between the gutted and ungutted groups. However, the sensory, microbiological, and chemical analyses indicated that the storage of the fish on ice produced from wild-thyme hydrosol had a substantial increase in shelf-life time when compared with barb individuals stored under traditional ice conditions.

Subsequently, Quitral et al. [24] checked the effect of aqueous extracts of oregano (*O. vulgare*) or rosemary (*R. officinalis*) leaves when included in icing medium used for the chilled storage of Chilean jack mackerel (*Trachurus murphyi*). Throughout a 23-day storage, a substantial antioxidant effect was detected in fish kept under both plant-extract icing systems, due to peroxide and thiobarbituric acid reactive substance formation. Additionally, the employment of such icing systems led to lower values for pH value and total volatile amine formation. Furthermore, the plant extract presence in the chilling medium led to lower free fatty acid formation (i.e., a decrease of lipid hydrolysis development).

The effect of an icing system including an aqueous rosemary (*R. officinalis*) extract on the rancidity stability and biogenic amine formation was studied in sardines (*Sardinella aurita*) during chilled storage [25]. As a result, storage of sardines in ice containing the rosemary extract led to a shelf-life time of 15 days, while a 10-day time was observed for fish from the control batch. Improved values were also obtained for chemical quality parameters (decreased values for pH, peroxides, and total volatile amines) compared with traditional icing. Furthermore, icing containing rosemary extract led to an inhibitory effect on biogenic amine formation, especially for histamine and putrescine.

Bensid et al. [26] studied the effect of ice containing thyme (*T. vulgaris*), oregano (*Origanum glandulosum*), or clove (*Syzygium aromaticum*) ethanolic extracts on the quality of chilled anchovy (*Engraulis encrasicholus*). According to sensory determination, anchovy stored in ice prepared with each plant extract revealed a shelf-life time of 12 days, while the batch stored in traditional ice provided a 9-day time. Additionally, the employment of plant-icing systems led to markedly lower counts of aerobic mesophiles and psychrotrophic bacteria in anchovy muscle, when compared with fish from the traditional ice batch; the same inhibitory effect was detected for total volatile amine and free fatty acid formation. Concerning lipid oxidation, a substantial antioxidant effect (decreased peroxide and thiobarbituric acid values) could be detected in fish kept under plant extract icing systems.

Viji et al. [27] tested the efficacy of ice containing 60% aq. ethanolic extracts of mint (*Mentha arvensis*) leaf or citrus (*Citrus aurantium*) peel for enhancing the quality of chilled Indian mackerel (*Rastrelliger kanagurta*). As a result, the presence of such plant extracts in the ice system reduced the generation of total volatile amines, trimethylamine, and free fatty acids in mackerel muscle during storage. Furthermore, a marked inhibition of lipid oxidation development (decreased peroxide and thiobarbituric acid reactive substance values) was also observed in fish stored in ice including plant extracts, when compared with samples from the control batch. Additionally, icing with plant extracts reduced the count of total viable bacteria. Sensory evaluation showed a shelf-life time of 13 days for fish stored under the conventional icing system, while values of 15 and 17 days were obtained for fish from the citrus peel and mint batches, respectively.

The effect on the shelf-life extension of chilled rainbow trout (*Oncorhynchus mykiss*) of ice including a 70% aq. ethanolic extract of reshgak (*Ducrosia anethifolia*) or including reshgak EO was analyzed by Tavakoli et al. [28]. Over a 20-day storage, both icing systems led to substantial lower bacterial activity (decreased total viable counts), as well as to lower levels in chemical quality indices (values of peroxides, thiobarbituric acid reactive substances, free fatty acids, and total volatile amines) when compared with fish from the control batch. According to sensory evaluation, fish stored in ice containing reshgak EO provided the longest shelf-life time (>16 days), while fish corresponding to the reshgak extract and traditional ice revealed values of 16 and 12 days, respectively.

**Figure 1:**
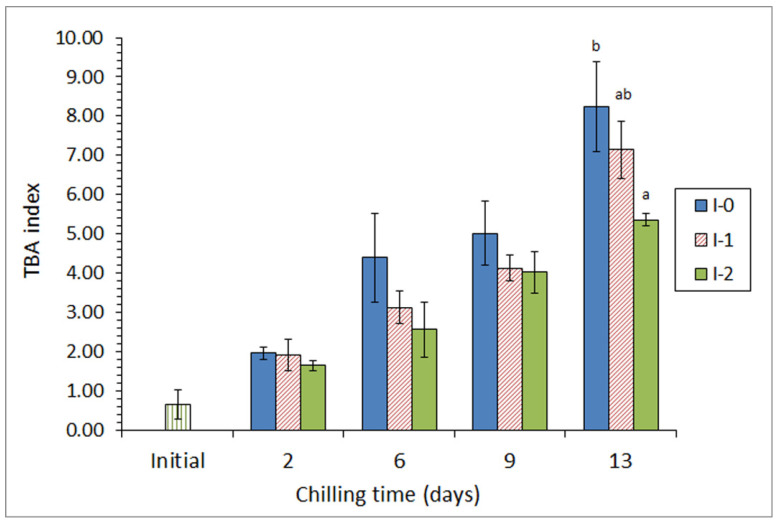
Evolution of thiobarbituric acid (TBA) index * in chilled chub mackerel subjected to different icing conditions **. * Average values of three independent determinations (*n* = 3). Standard deviations are indicated by bars. Average values accompanied by different letters indicate significant differences (*p* < 0.05). ** Icing conditions: I-0 (traditional ice; control), I-1 (ice including a low concentration of quinoa extract), and I-2 (ice including a high concentration of quinoa extract). Adapted from Miranda et al. [29].

Two different concentrations of 86% aq. ethanolic extracts of lyophilized quinoa (*Chenopodium quinoa*) were included in the icing medium employed during the chilled storage of Atlantic chub mackerel (*Scomber colias*) [29]. Their effect on fish quality was studied over a 13-day storage. As a result, a lower secondary (formation of thiobarbituric acid reactive substances; Figure 1) and tertiary (fluorescence values) lipid oxidation development was detected in fish from the most concentrated quinoa batch. Similarly, a lower lipid hydrolysis development (determination of free fatty acid content and lipolytic bacteria counts) and a decrease in the pH and trimethylamine levels were observed in the mentioned batch. Sensory analysis showed that fish specimens from this batch were the only samples still acceptable at the end of storage time; notably, quality limiting descriptors were skin, eyes, and external odor.

An 86% aq. ethanolic saponin-free quinoa extract was added to the icing system employed for the chilling storage of a lean (megrim, *Lepidorhombus whiffiagonis*) and a fatty (Atlantic chub mackerel, *S. colias*) fish species [30]. For a 13-day storage, an inhibitory effect of the presence of quinoa extract in the icing system was proven on the microbial development in megrim (decrease of aerobe counts, pH, and free fatty acid value) and mackerel (decrease of aerobe, psychrotroph, proteolytic, and lipolytic counts and of pH and free fatty acid values), when compared with samples from the control batch of each fish species.

Aqueous extracts of *Garcinia indica* or *Garcinia cambogia* were included in the icing system employed for the chilled storage of Indian mackerel (*R. kanagurta*) [31]. As a result, both kinds of garcinia extracts reduced the values of trimethylamine, total volatile amines, peroxides, thiobarbituric acid reactive substances, and total viable counts when compared with fish stored under control ice. Remarkably, the sensory evaluation showed, shelf-life times of 15, 21 and 24 days for fish kept under traditional icing, ice containing *Garcinia indica* extract, and ice containing *Garcinia cambogia* extract, respectively.

## 3. Low-Molecular-Weight Organic Acids in the Icing Medium: Effect on Chilled Seafood Quality

### 3.1. General Aspects of Low-Molecular-Weight Organic Acids

Low-molecular weight organic acids (i.e., acetic, lactic, citric, malic, and ascorbic) are naturally occurring compounds present in many food of plant origin, as well as produced during the fermentation of food [32]. Such compounds have the common denominator of having carbon in their structure, exist in two basic forms (pure acids or buffered acids), and have 10 or less carbons in their structure; being distinguished from fatty acids that have straight, even-number carbon chains of 4 to 24.

Such organic acids have a long history of being utilized as food additives (i.e., acidulants, flavorants) and preservatives for preventing food deterioration and extending the shelf-life time of perishable food ingredients [33]. Today, they are receiving increasing attention as minimal processing strategies, because they are easily attained, have a low commercial cost, and can be used in food at a wide range of permitted concentrations; these having to be declared on food labels [34]. Consumers generally accept the use of organic acids and their salts in food, since they regard organic acids as food-grade compounds, and recognize their use in households as flavorings or natural food acidulants from ancient times [33]. Such compounds have been added directly to seafood or have been included in aqueous solutions in which marine products are dipped for a certain time before subsequent storage or processing [35,36]. Nevertheless, fish traders are required to verify the correct employment and addition of such organic acids before presenting their products to consumers [37].

The antimicrobial activity of organic acids is explained on the basis of being soluble in lipids in their undissociated forms, which allows them to cross the microbial membrane into the microbial cytoplasm, where the acids tend to dissociate and deliver hydrogen ions and the corresponding anions (citrate, ascorbate, etc.) [38]. As a result, microorganisms are forced to export the excess hydrogen to maintain a physiological pH inside the cell, which is an energy-depleting process that limits bacterial growth. Otherwise, the excess hydrogen ions in the cytoplasm may cause the pH to decrease to levels that are incompatible with bacterial growth [39]. Antimicrobial efficacy would depend on the pH value, water activity, moisture, fat, nitrite, and salt content of the product, as well as the storage conditions (temperature, packaging atmosphere) [11,40].

Some organic acids have also proved to have a marked effect on lipid oxidation inhibition in food in general. Thus, autoxidation due to the presence of heavy metals is inhibited by substances inactivating the catalytic action of these metals by forming complexes or chelating them. Among the natural organic acids, ascorbic acid and citric acid and their salts are well-known chelators in biological systems [41,42]. Thus, a previous dipping treatment using both acids was shown to increase the rancidity stability (primary, secondary, and tertiary lipid oxidation development) of fish fillets [43] and whole fish [44]. Ascorbic acid (i.e., vitamin C) is highly susceptible to oxidation during food processing in general. Thus, the oxidation of ascorbic acid has been detected as one of the earliest events during seafood chilled storage; this behavior being explained on the basis of its low reduction potential [5].

### 3.2. Quality Enhancement of Chilled Seafood by Including Natural Low-Molecular-Weight Organic Acids in Ice

**Figure 2:**
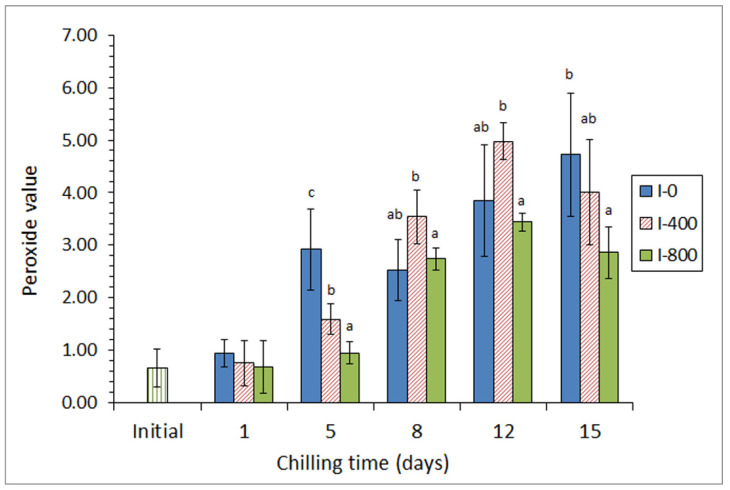
Peroxide formation * in chilled hake subjected to different icing conditions **. * Average values of three independent determinations (*n* = 3). Standard deviations are indicated by bars. Average values accompanied by different letters indicate significant differences (*p* < 0.05). ** Icing conditions: I-0 (traditional ice; control), I-400 (ice including a low concentration of the acid mixture), and I-800 (ice including a high concentration of the acid mixture). Adapted from García-Soto et al. [45].

In a first approach [45], a chilling strategy employing a mixture of different preservative organic acids (ascorbic, citric, and lactic) in the icing medium was applied for the chilled storage of three lean fish species (hake, *Merluccius merluccius*; megrim, *L. whiffiagonis*; angler, *Lophius piscatorius*). For this, two different concentrations of the acid mixture were applied for a 15-day chilling storage trial. A partial inhibition of primary (peroxide detection, Figure 2), secondary (thiobarbituric acid reactive substances assessment), and tertiary (determination of the fluorescence ratio) lipid oxidation development was obtained in fish species for the highest concentration of the acid mixture; this result was accompanied by a shelf-life enhancement in all samples tested, as well as by an inhibitory effect on free fatty acid formation.

Later, the same authors analyzed the effect of two concentrations of such preservative organic acid (ascorbic, citric, and lactic) mixture on the microbial activity development during chilled storage (up to 12–15 days) in the same three lean fish species [46]. Lower counts of mesophiles were found for hake and megrim samples from both treated batches when compared with the control. In the case of angler, lower counts of mesophiles, psychrotrophs, and proteolytic microorganisms were found for samples stored under icing conditions including the highest concentration of the acid mixture. Both treated megrim batches exhibited lower pH values than the control batch, and this result was also observed in the angler batch with the most concentrated acid mixture. Sensory evaluation indicated that a longer shelf-life time was obtained in all three fish species when stored in the most concentrated acid mixture condition.

The oxidative stability of a chilled medium-fat fish species (horse mackerel, *Trachurus trachurus*) was determined when two concentrations of a mixture of natural organic acids (citric, ascorbic, and lactic) were incorporated in the icing medium employed [47]. After a 13-day storage, the determination of peroxide values and content of thiobarbitiric acid-reactive substances indicated that the addition of the acid mixture to the chilling system inhibited the lipid oxidation development. Sensory evaluation showed that fish samples from the most concentrated acid mixture batch provided a longer shelf-life time. However, the presence of acids in the icing system had no impact on the lipid hydrolysis development (i.e., free fatty acid formation).

Later, Sanjuas-Rey et al. [48] investigated the effect of including a mixture of organic acids (citric, ascorbic, and lactic) in the icing medium employed for a 13-day chilled storage of a fatty fish species (Atlantic mackerel, *S. scombrus*). Analysis of fish muscle quality indicated a lower bacterial growth (determination of aerobe, anaerobe, psychrotroph, *Enterobacteriaceae*, lipolytic, and proteolytic counts) in samples subjected to storage in the organic acid-icing system, as well as lower levels for two chemical indices related to microbial activity development (assessment of total volatile amine and trimethylamine contents).

In order to enhance the quality of chilled blue whiting (*M. poutassou*), a natural organic acid-mixture, including ascorbic, citric, and lactic acids was applied in a two-step processing strategy: (i) as an aqueous dipping medium prior to chilling storage, and (ii) included in the flake ice employed as the chilling system [49]. As a result of this two-step treatment, an inhibition of the microbial and biochemical mechanisms related to the quality loss was recorded. Concerning microbial activity, the aerobe and psychrotroph counts in treated blue whiting showed lower ranges than the control fish. The sensory evaluation indicated that the treated fish were still acceptable at day 9, while control fish were rejected at this time. The lipid hydrolysis development of fish muscle (determination of free fatty acid content) proved to be more limiting of fish quality than lipid oxidation (detection of peroxide and thiobarbituric acid-reactive substance formation).

Three combinations of two natural organic acids, citric acid, and lactic acid were incorporated in the icing systems employed for the chilling storage of European hake (*M. merluccius*) [50]. Thus, the resulting flake icing systems were prepared with 0.075%/0.050% (C-75 batch), 0.125%/0.050% (C-125 batch), and 0.175%/0.050% (C-175 batch) citric acid/lactic acid, respectively, and comparatively analyzed for microbial activity development and lipid damage. Lower microbial counts were found in the C-175 batch for all microbial groups investigated when compared with fish from the control batch. All treated batches showed a decrease of the trimethylamine value, this effect being greater as the citric acid concentration in the ice increased. A marked inhibitory effect on fluorescent compound formation (i.e., tertiary lipid oxidation) due to the presence of organic acids in the icing systems was also observed at advanced storage times. Additionally, an extension of the shelf-life time was detected in fish from the C-125 and C-175 batches.

Later, the same group analyzed the effect of aqueous solutions of two different concentrations of citric acid and lactic acid for the chilled storage of a flat fish species (megrim, *L. whiffiagonis*) [51]. Throughout a 13-day chilled storage, a lower bacterial growth was detected according to microbiological count assessment (aerobe and psychrotroph values) and chemical determinations related to microbial activity (trimethylamine and pH levels). Furthermore, the sensory evaluation led to a shelf-life increase in treated fish when compared with the control batch. Thus, the control fish showed a shelf-life of 9 days, while all acid-iced fish were still acceptable at the end of the experiment. Concerning lipid damage, an inhibitory effect on the fluorescent compound formation (tertiary lipid oxidation) was observed in the acid-iced samples.

In order to enhance the quality of European hake (*M. merluccius*) and megrim (*L. whiffiagonis*) during on-board chilling storage, an aqueous solution including citric and lactic acids was applied as an icing medium [52]. The effect of the acid mixture in the icing system was analyzed after 9, 12, and 15 days of on-board storage. As a result, a lower bacterial growth was detected, according to microbial activity determination (formation of aerobe, anaerobe, psychrotroph, proteolytic, and *Enterobacteriaceae* counts) and trimethylamine level assessment. Furthermore, a substantial inhibitory effect on autolysis development (K value determination) in the hake was also detected. Finally, an enhancement of sensory scores (eyes, external odor, and gills) in both acid-iced species was detected.

## 4. Macroalga-Extract Presence in the Icing Medium: Effect on Chilled Seafood Quality

### 4.1. General Aspects of Macroalga-Extract Compounds

Marine macroalgae are multicellular photosynthetic organisms that contribute to nearly 10% of total marine productivity [53]. Seaweeds, the popular term for marine macroalgae, are classified based on anatomy, pigmentation, morphology, chemical composition, and other characteristics as green (chlorophyte), brown (phaeophyte), and red (rhodophyte) algae. Recent decades have seen increasing attention given to macroalgae metabolites in industries from different fields (textile, fuel, plastics, paint, varnish, cosmetics, pharmaceutical, and food) [54]. Thus, more than 15,000 primary and secondary metabolites from different pathways have been reported for macroalgae, and different applications have been assigned to them [55].

For centuries, marine algae have been included in the Asian diet, especially in countries like China, Japan, and Korea. Interestingly, their consumption has increased in recent years in Western countries due to the search for new sustainable sources of healthy food and natural products [56]. Seaweeds have been shown to be a good source of beneficial constituents such as lipids, vitamins, trace minerals, dietary fiber, and amino acids [57]. The lipid composition of marine algae has received considerable interest, due to the high content of polyunsaturated fatty acids, especially C18:3n-3 (linolenic acid), C18:4n-3 (stearidonic acid), C20:4n-6 (araquidonic acid), C20:5n-3 (eicosapentaenoic acid), and C22:6n-3 (docosahexaenoic acid) acids.

Notably, algae in general are known to be exposed to a combination of high oxygen concentration and light. The lack of structural damage to their organs has led to the consideration that their protection against damage arises from their content of preservative substances [58]. Marine macroalgae have been reported to contain a wide variety of chemical constituents with potential antioxidant and antimicrobial activities, suitable to being applied to seafood and food in general [59,60,61]. Bioactive compounds, such as polyphenols, pholorotannins, terpenes, polysaccharides, peptides, chlorophylls, and carotenoids, have been isolated from different algae species and found to be responsible for this preservative behavior [62]. Thus, a wide range of studies have reported the antioxidant and antimicrobial influence of crude extracts from seaweeds using simple and fast in vitro assays [63,64]. In addition, algae are considered food or food ingredients [65], so their use in food technology in general should not constitute any hazard to health. However, on the basis of possible toxicological issues resulting from marine pollutants (namely, heavy metals), fish traders ought to verify the algae composition before addition to seafood and food in general [66,67].

### 4.2. Quality Enhancement of Chilled Seafood by Including Macroalga Extracts in Ice

As a first approach, two different concentrations of ethanolic extracts of alga *Fucus spiralis* were included in an icing medium used for a 14-day chilled storage of megrim (*L. whiffiagonis)* [68]. Compared with the counterpart control batch, a substantial inhibition of microbial activity (determination of aerobe, psychrotroph, proteolytic bacteria, and lipolytic bacteria counts; assessment of pH and trimethylamine formation) was detected in fish from both treated batches, especially in the most concentrated batch. Regarding lipid oxidation, a lower formation of interaction compounds (assessment of fluorescent compound formation) in megrim samples from the most concentrated batch was also observed.

**Figure 3:**
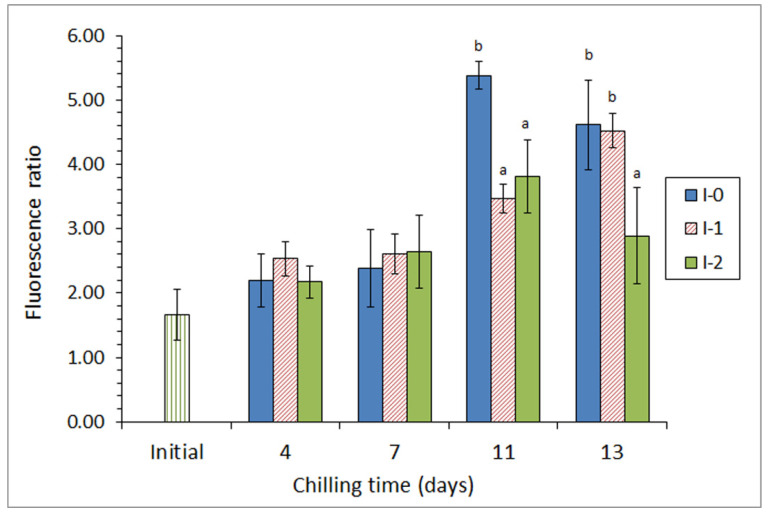
Evolution of the fluorescence ratio * in chilled megrim subjected to different icing conditions **. * Average values of three independent determinations (*n* = 3). Standard deviations are indicated by bars. Average values accompanied by different letters indicate significant differences (*p* < 0.05). ** Icing conditions: I-0 (traditional ice; control), I-1 (ice including a low concentration of the alga extract), and I-2 (ice including a high concentration of the alga extract). Adapted from García-Soto et al. [69].

Subsequently, the same authors [69] included ethanolic extracts of a different brown macroalga (*Bifurcaria bifurcata*) in the icing medium employed for the chilled storage of megrim (*L. whiffiagonis*). Two different concentrations of this macroalga extract were tested in a 13-day storage. For alga *F. spiralis*, a marked microbial activity inhibition was observed (detection of aerobes, psychrotrophs, lipolytic bacteria, proteolytic bacteria, and *Enterobacteriaceae* counts), as well as lower levels of pH and trimethylamine being detected; this effect being especially relevant in the most concentrated batch. Related to lipid damage, a lower lipid hydrolysis development was proven and a lower level of tertiary lipid oxidation compounds was detected in fish from both batches including alga extracts, when compared with fish stored under the control conditions (Figure 3); as for the microbial inhibition, this effect was found to be stronger in fish from the most concentrated batch.

Arulkumar et al. [70] incorporated a methanolic red alga *Gracilaria verrucosa* extract at two concentrations in an icing system employed for the chilled storage of Indian mackerel (*R. kanagurta*). During a 15-day storage trial, an inhibitory effect on microbial activity (counts of mesophilic and psychrophilic bacteria) and chemical parameters related to microbial activity (determination of pH, trimethylamine, total volatile amines, and biogenic amines) was observed. Sensory determination showed a shelf-life time of 11 days for the control fish, while treated mackerel were still acceptable at the end of the study. The preservative effect found for alga extracts was explained on the basis of the subsequent identification in the methanol extracts of preservative agents, such as butylated hydroxytoluene, sulphurous acid, 1,2-propanediol, benzene acetic acid, cyclononasiloxane, and tetracosamethylcyclo-dodecasiloxane [70].

Ethanolic extracts of alga *Undaria pinnatifida* were included in the icing system used during the 9-day chilled storage of megrim (*L. whiffiagonis*) [71]. The presence of the alga extract in the icing medium led to an inhibitory effect on lipid hydrolysis development (free fatty acid detection). Notably, all fish batches revealed a low primary (i.e., peroxides) and secondary (thiobarbituric acid reactive substances) lipid oxidation compound formation. However, the determination of fluorescent compound formation (tertiary lipid oxidation compounds) showed lower average scores for the treated megrim at advanced storage periods (6–9 days), so that a partial inhibition of lipid oxidation development could be concluded as a result of the presence of the alga extract in the icing medium. The inhibitory effect on lipid damage was justified by a marked polyphenol content and a good antioxidant capacity measured by DPPH assay. Additionally, the treated fish provided lower counts of aerobes, proteolytic, and lipolytic bacteria, proving an inhibitory effect on microbial activity development.

The effect of different kinds of extracts (aqueous and ethanolic) of alga *B. bifurcata* when incorporated in the icing system employed for the chilled storage of hake (*M. merluccius*) was comparatively studied by Miranda et al. [72]. During a 13-day storage, a substantial inhibitory effect on microbial activity could be observed as a result of including the aqueous (decrease of psychrotrophic and lipolytic levels and pH value) and ethanolic (decrease of psychrotrophic and lipolytic contents) extracts, when compared with fish stored under control conditions. Furthermore, both extracts led to a marked inhibition of free fatty acid formation (lipid hydrolysis development), which was found to be greater in fish from the ethanolic-extract batch. Concerning the lipid oxidation development, a marked inhibitory effect on the formation of secondary compounds (thiobarbituric acid reactive substances) was noticed in hake with both alga extracts; however, a higher peroxide formation was obtained in hake from the ethanolic extract batch.

Different kinds of extracts (aqueous, ethanol, and ethanolic-aqueous) of alga *F. spiralis* were included in the icing medium employed for the chilled storage of hake (*M. merluccius*) [73]. The effects of such extracts on hake quality were comparatively analyzed during a 13-day period. It could be observed that the presence of alga ethanolic extracts (alone or combined with aqueous extract) in ice led to a marked antimicrobial effect against aerobe, psychrotroph, proteolytic, and lipolytic bacteria. Furthermore, an inhibitory effect on both batches was also detected concerning lipid oxidation development (i.e., secondary and tertiary compound formation). Notably, lower mean values of tertiary oxidation compound formation were observed in fish corresponding to the combined (i.e., aqueous–ethanolic) batch throughout the whole storage period.

An ethanolic-aqueous extract of alga *Cystoseira compressa* was included in the icing system employed for the 11-day chilled storage of a medium-fat fish species (horse mackerel, *T. trachurus*) [74]. Determination of the microbial group counts (aerobe, psychrotroph, proteolytic, lipolytic, and *Enterobacteriaceae* bacteria) showed an inhibitory effect of microbial activity in fish muscle as a result of the presence of the alga extract in the icing medium. Additionally, chemical determinations related to microbial activity (pH and trimethylamine levels) also showed a beneficial preservative effect. Concerning lipid damage, a decrease of lipid hydrolysis (free fatty acids assessment) and oxidation (fluorescent compounds detection) development in horse mackerel muscle was also detected, resulting from the presence of the *C. compressa* extract in the icing system.

Barbosa et al. [75] incorporated three different concentrations of an ethanolic-aqueous extract of red alga *Gracilaria gracilis* as a source of preservative compounds to be applied for the 9-day chilled storage of hake (*M. merluccius*). An inhibitory effect on lipid oxidation (formation of tertiary compounds) and microbial activity (trimethylamine assessment) was observed in fish from batches including the two most concentrated alga batches. Contrarily, a definite effect on lipid hydrolysis (free fatty acids detection) could not be concluded in any of the treated-fish batches. Interestingly, the application of these natural compounds was not detrimental, and positively assessed by panelists involved in the storage trials reviewed in this paper.

## 5. Presence of Seafood By-Product Extracts in the Icing Medium: Effect on Chilled Seafood Quality

### 5.1. General Aspects of Seafood By-Products

Fishing and aquaculture play an important role in human societal development. Nowadays, annual seafood production contributes over 170 million tons of fish and shellfish worldwide [76]. Nonetheless, only 50 to 60% of the total catch is used for direct human consumption, seafood processing being considered one of the main sources of by-products (heads, blood, viscera, skin, tails, etc.) [77]. Remarkably, the highest concentrations of high-added-value compounds, such as minerals, lipids, enzymes, pigments, vitamins, amino acids, polysaccharides, and proteins are often present in the body parts of marine organisms that are commonly discarded [78,79]. Additionally, a considerable volume of undesired products is obtained, constituting an important source of environmental contamination unless efforts for their recovery are carried out, while their commercial value can be enhanced via extraction of valuable constituents [80,81].

At present, the majority of these by-products are sent to fish meal plants, where fish meal and fish oil are produced. Fish meal is by far the most valuable non-edible commodity produced from marine by-products; thus, its global production reached a level of 18 million tons in 2018 [82]. Furthermore, non-nutritional applications are nowadays also attracting attention, as in the case of chitin and chitosan, leather, pharmaceuticals, cosmetics, fine chemicals, collagen, gelatin, and others [83]. Meanwhile, a great deal of attention is being given to converting seafood by-products into sources of bioactive compounds that could be employed in seafood technology as preservative substances and in human nutrition [80,84].

### 5.2. Quality Enhancement of Chilled Seafood by Including by-Product Extracts in Ice

Aqueous solutions, including acetic acid–ethanol extracts of jumbo squid (*Dosidicus gigas*) skin (JSS), were tested at three different concentrations as icing media [85]. Their effect on the quality evolution of a fatty fish species (mackerel, *S. scombrus*) during chilled storage was analyzed. A marked inhibition of microbial activity was determined in the fish batch under the icing condition including the highest JSS extract concentration. Furthermore, fish specimens corresponding to batches including any of the JSS extract concentrations applied led to lower proteolytic counts and pH values than the control mackerel. A substantial shelf-life extension in chilled fish stored in ice, including the highest JSS concentration was detected; specimens from these batches were still edible after a 13-day storage, while all other mackerel batches were considered inedible at that time. The resulting microbial inhibition was explained by the presence in the ice of lipophilic compounds (i.e., ommochrome pigments) obtained by the acetic acid–ethanol extraction of JSS.

The same authors [86] employed aqueous solutions including acetic acid–ethanol extracts of JSS at two different concentrations as icing media during the chilled storage of a lean fish species (hake, *M. merluccius*). As a result, a substantial inhibition of microbial activity (determination of aerobe, psychrotroph, *Enterobacteriaceae*, and proteolytic bacteria counts; pH and trimethylamine assessment) was obtained in fish from the icing batch including the highest JSS concentration. Additionally, hake specimens in this icing condition displayed an inhibitory effect on free fatty acid formation, while no effect could be concluded for lipid oxidation (assessment of peroxide and thiobarbituric acid reactive substances). Evaluation of sensory quality (skin and mucus development; eye and gill appearance; texture; external odor; raw and cooked flesh odor; flesh taste) indicated a shelf-life extension of chilled hake stored in ice including the highest JSS concentration. According to previous related research, ommochrome pigments (i.e., lipophilic-type compounds) were found to be responsible for this preservative effect.

## 6. Final Remarks and Future Trends

In agreement with actual life-style needs, advanced developments in the production, distribution, and retailing of food are continuously being offered to consumers. Related to the constant need for high-quality fresh seafood, the inclusion of natural preservative compounds in the icing system can be considered as a highly profitable strategy to obtain nutritious, attractive, and safe products. With the aim of increasing the practical and commercial employment of this technological strategy, the following recommendations and aspects can be taken into account in order to offer the consumer an optimally fresh product:

*Degree of quality of raw material:* With traditional chilled storage, preliminary on-board and on-land handling should be carried out as carefully as possible, so that the slaughter step is achieved under minimally stressing conditions and so that the cold chain is maintained from catching/harvesting till delivering to the consumer.

*Safety of the natural preservative compounds or extracts:* Natural substances might also have health-associated risks that should be addressed. Before practical and commercial employment, preservative compounds or extracts for inclusion in the icing system ought to agree with international regulations concerning health risks.

*Optimization of the presence of preservative compounds or extracts in ice:* According to most research carried out to date, a different response is produced depending on the kind of marine species (size, fat content, general composition, skin resistance, wild or cultivated, etc.) and other biological aspects (capture season, maturity, sex, eating state, etc.). Therefore, further studies on ascertaining optimum levels of preservative compound content for inclusion in ice preparations ought to be developed.

*Selection of the type of preservative compounds or extracts:* The type of natural extract (aqueous, ethanolic, etc.) included in the ice ought to be optimized in each case (namely, type of species) to enhance the sensory and nutritional values of the fresh seafood; rather than extrapolating the findings made with any other products. For this purpose, the suitability of each kind of preservative extract or compound (more or less lipophilic or hydrophilic) for each marine fish or invertebrate species ought to be analyzed and optimized.

*Search for synergistic effects with other technologies:* It is commonly accepted that the combination of preservatives and other stress factors is one of the most promising strategies for the prevention of seafood damage and of food in general. The search for synergistic combinations with other advanced technologies, such as slurry ice and super-chilling in general, non-thermal technologies (i.e., different kinds of irradiation, high pressure processing), or addition of other natural preservatives, must be intensified to increase the degree of quality, as well as the shelf-life time of the resulting chilled seafood.

*Consumer requirements:* Current and future preparations of seafood ought to focus on the development of attractive products that fulfil consumer expectations for odor, color, taste, flavor, and general appearance. A special stress ought to be placed on the possible effect of the presence of preservatives in ice on the sensory characteristics expected in fresh seafood. The content of preservative compounds in ice ought to be optimized so that the raw product sensory characteristics are not modified in the resulting chilled seafood.
